# Fat- and sugar-induced signals regulate sweet and fat taste perception in *Drosophila*

**DOI:** 10.1016/j.celrep.2023.113387

**Published:** 2023-11-06

**Authors:** Yunpo Zhao, Emilia Johansson, Jianli Duan, Zhe Han, Mattias Alenius

**Affiliations:** 1 Department of Molecular Biology, Umeå University, Umeå, Sweden; 2 Center for Precision Disease Modeling, University of Maryland School of Medicine, Baltimore, MD, USA; 3 Present address: Center for Precision Disease Modeling, University of Maryland School of Medicine, Baltimore, MD, USA; 4 Lead contact

## Abstract

In this study, we investigate the interplay between taste perception and macronutrients. While sugar’s and protein’s self-regulation of taste perception is known, the role of fat remains unclear. We reveal that in *Drosophila*, fat overconsumption reduces fatty acid taste in favor of sweet perception. Conversely, sugar intake increases fatty acid perception and suppresses sweet taste. Genetic investigations show that the sugar signal, gut-secreted Hedgehog, suppresses sugar taste and enhances fatty acid perception. Fat overconsumption induces unpaired 2 (Upd2) secretion from adipose tissue to the hemolymph. We reveal taste neurons take up Upd2, which triggers Domeless suppression of fatty acid perception. We further show that the downstream JAK/STAT signaling enhances sweet perception and, via Socs36E, fine-tunes Domeless activity and the fatty acid taste perception. Together, our results show that sugar regulates Hedgehog signaling and fat induces Upd2 signaling to balance nutrient intake and to regulate sweet and fat taste perception.

## In brief

Zhao et al. discover that sugar and fat balance each other’s taste perception. They demonstrate that fat inhibits fatty acid taste and increases sweet taste via Upd2 signaling. They also reveal that gut-secreted Hedgehog inhibits sweet and increases fatty acid taste downstream of sugar. Thus, these signals balance taste with requirement.

## INTRODUCTION

Animals choose their food according to physiological requirements, influenced by factors such as food availability, diet nutrient content, and reproductive status. In *Drosophila*, feeding choices are affected by sex, mating status, and nutrient levels in the diet. Male and virgin female flies favor sugar over yeast, while mated females shift to preferring yeast.^1,2^ Low protein intake leads to a compensatory yeast appetite in mated females and is mediated by direct neuronal nutrient sensing^3–5^ and increased taste perception.^6^ Hence, taste perception shapes food selection and connects to metabolic and energy needs.

Apart from protein, the remaining macronutrients—fat and sugar—emerge as attractive tastes for animals. However, excessive consumption of sugars and specific fatty acids, like hexanoic acid and octanoic acid, can be toxic to flies and must be regulated.^7^ In *Drosophila*, sugars are detected by eight taste receptors (*Gr5a, Gr61a*, and *Gr64a-f*), triggering sweet taste neurons on the labellum, pharynx, and legs.^6,8–11^ High sugar intake reduces mated starved females’ taste response to sugar,^12–14^ and male flies likewise diminish their perception of sweetness and preference for sweet foods with increased sugar intake.^15^

In *Drosophila*, fatty acids are detected by ionotropic receptors (*IR25a, IR56d*, and *IR76b*), as well as the gustatory receptor *GR64e* on sweet taste neurons associated with positive valence.^16–19^ Additionally, gustatory receptors (*Gr32a, Gr33a,* and *Gr66a*) involved in bitter taste perception associate high fatty acid concentrations with negative valence.^16,20^ The coactivity of sweet taste neurons for both fat and sugar implies that sugar consumption might influence fatty acid perception and potentially impact the uptake of fatty acids.

We recently demonstrated that sugar consumption induces the gut to secrete Hedgehog (Hh) into the circulatory system of *Drosophila*.^15^ We have also shown that autocrine Hh signaling in olfactory sensory neurons regulates odorant receptor transport and odor perception.^21^ Intriguingly, the gut-derived Hh protein enters the lymph of both taste and olfactory sensilla, where it suppresses the autocrine Hh signaling through an unidentified mechanism. The gut-secreted endocrine Hh signal regulates ol-factory sensitivity, sweet taste perception, and food preferences, potentially affecting the perception of fatty acid taste as well.

Across phyla, fat intake is known to induce leptin expression and secretion,^22–26^ where it regulates food intake, thermogenesis, energy expenditure, and the homeostasis of glucose/lipid metabolism.^27^ Vertebrate leptin regulates body mass via a negative feedback loop connecting adipose tissue to the hypothalamus, hippocampus, and brain stem.^28,29^ In flies, one of three leptin orthologues, *unpaired 2* (*Upd2*), is released in response to fat from the fat body to regulate lipid metabolism and insulin secretion from the insulin secreting cells.^24^ This suggests that Upd2 acts as an adipose feedback signal, but it is unclear whether it can also regulate fatty acid taste perception.

Here, we present evidence that fat overconsumption suppresses fatty acid taste perception, much like sugar suppresses sweet taste. This suppression is mediated by the fat-induced secretion of Upd2 from the fat body. Upd2 activates Dome receptors on sweet taste neurons, leading to the suppression of fatty acid taste perception and in parallel the activation of JAK/STAT signaling, enhancing sweet taste. Furthermore, sugar-induced Hh secretion from the gut enhances fatty acid perception, conforming to the previously demonstrated suppression of sweet perception from exposure to excessive dietary sugar levels. Our results together thus show that in parallel to the inhibition of acute feeding by insulin, an unbalanced fat or sugar intake regulates taste perception in a manner that rebalances sugar and fat nutrient consumption rather than reducing total caloric intake.

## RESULTS

### Dietary sugar increases fatty acid taste perception

**Figure 1.**
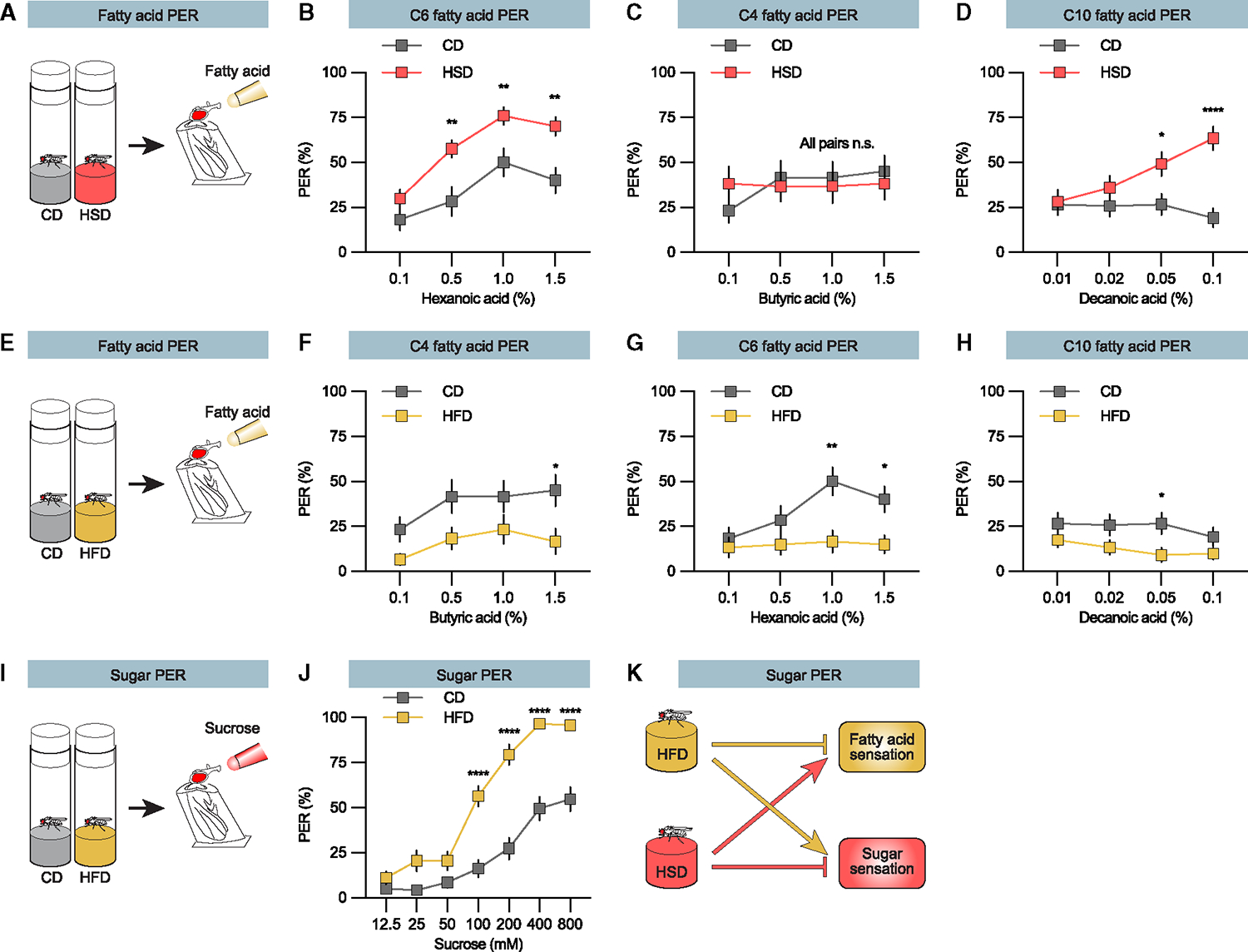
Reciprocal regulation of taste perception by dietary sugar and fat (A, E, and I) Schematic illustration of the PER assays.^44–46^ Newly eclosed flies were fed the indicated diets for 4 days, mounted, and stimulated with either fatty acid (A and E, yellow tip) or sucrose (I, red tip) solutions. (B–D) PER of flies fed a high-sugar diet (HSD, red line) or respective control diet (CD, gray line), when stimulated with (B) hexanoic acid (C6), (C) butyric acid (C4), and (D) decanoic acid (C10). n = 20 to 40 flies. (F–H) PER of flies fed a high-fat diet (HFD, yellow) or respective control diet (CD, gray line), when stimulated with (F) butyric acid (C4) on butyric acid, (G) hexanoic acid (C6), and (H) decanoic acid (C10). n = 20 to 40 for each group. (J) Sugar PER for flies fed an HFD (yellow line) compared to CD (gray line). n = 39 for each group. (K) Model for taste regulation by the HFD and HSD. Data are presented as means ± SEM. PER data were non-normally distributed. Statistical analysis was performed via Mann-Whitney tests. *p < 0.05; **p < 0.01; ****p < 0.0001. For fly food recipes, see Table S1.

Sweet taste neurons respond to both sugars and fatty acids.^16^ Dietary sugar suppresses the sweet taste neuron response to sugar.^12,14,15,30^ Thus, it is possible that sugar overconsumption can regulate fat taste perception. To explore this, we assessed fatty acid perception by observing proboscis extension response (PER) toward hexanoic acid (Figures 1A and 1B). Flies on a balanced low-fat and -sugar control diet (CD, 6% sucrose) exhibited robust and frequent extensions in response to hexanoic acid (Figure 1B). When newly eclosed flies were shifted to a high-sugar diet (HSD, 34% sucrose) for 4 days, the hexanoic acid PER increased (Figure 1B), suggesting that sugar overconsumption enhances the response to fatty acids. Flies differentiate the taste of fatty acids by their chain length.^31^ Expanding our analysis to include both short, four-carbon (C4, butyric acid) and long, ten-carbon (C10, decanoic acid) fatty acids showed that exposure to HSD increased PER to long fatty acids (Figures 1B–1D). There was a non-significant increase in PER to the short fatty acid at the lowest tested concentration suggesting that a possible change might be at even lower concentrations. Together, these results imply that sugar overconsumption shifts the sweet taste neurons response from sugars to fatty acids.

### Dietary fat suppresses fatty acid taste perception and increases sugar taste perception

To explore whether fat overconsumption also regulates sweet taste neuron function, we transitioned flies to a high-fat diet (HFD, 14% fat), calorie matched with the HSD (1,866 calories for HSD, 1,935 calories for HFD), and we assessed the PER for fatty acids (Figure 1E). Consistent with a regulatory role, the PER response for all fatty acids diminished as flies were exposed to the HFD (Figures 1F–1H). These findings suggest that fat, similar to sugar, also dampens its own taste perception in *Drosophila*.

To address if fat and sugar show a reciprocal regulation of sweet taste neurons, we again exposed flies to an HFD and now assessed the sugar PER (Figure 1I). Supporting reciprocal regulation between the two macronutrients, the HFD did indeed increase the sugar PER responses (Figure 1J). These findings show that sugar and fat jointly modulate taste (Figure 1K), not merely to minimize caloric intake but to shift taste perception toward the underfed nutrient.

### Sugar-induced Hh regulates both sweet and fatty acid taste perception

**Figure 2.**
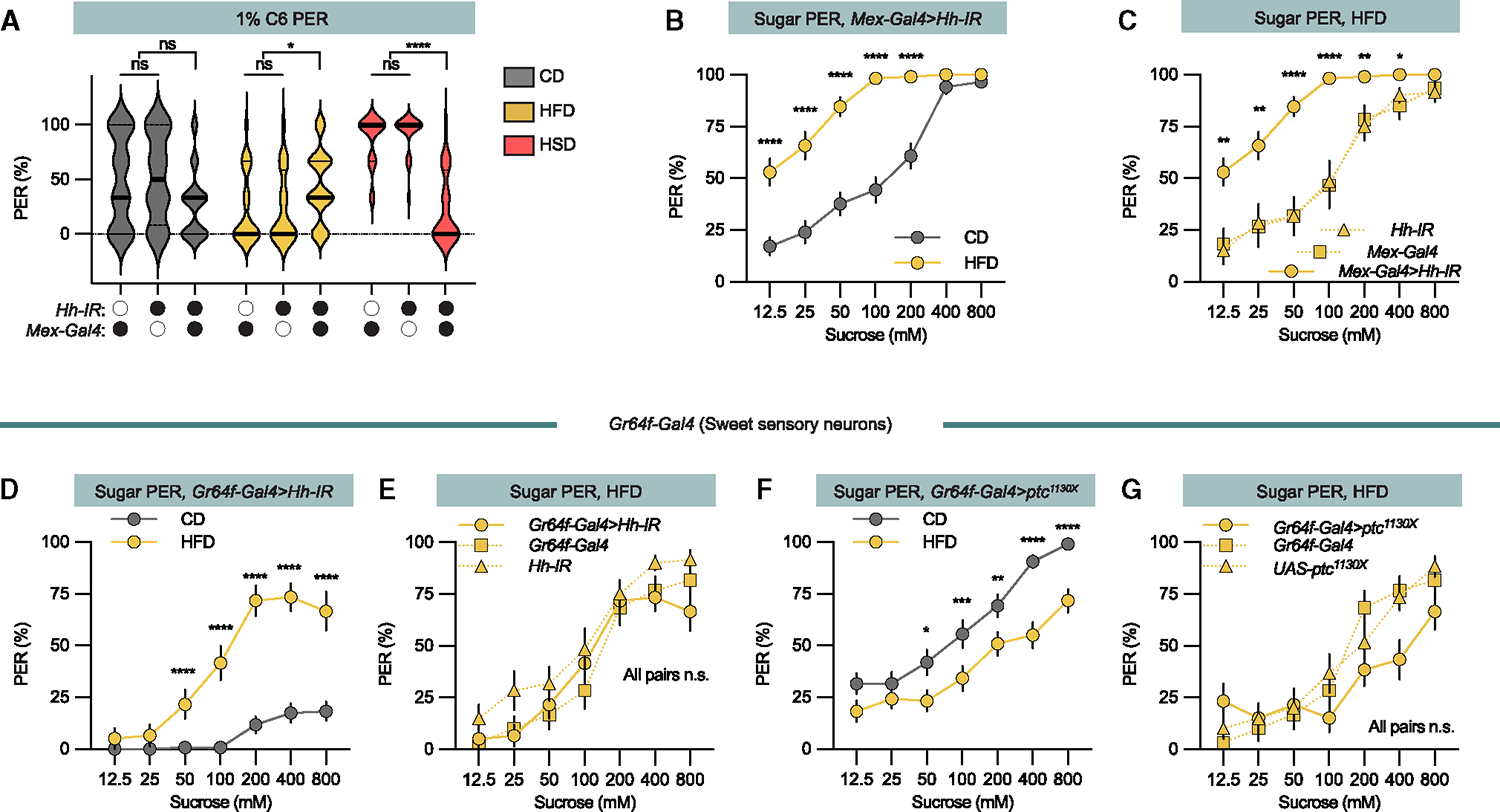
Hh regulates both sweet and fatty acid taste perception (A) 1% C6 PER (flies were stimulated with 1% hexanoic acid [HA]) of *Mex-Gal4>Hh-IR* and genetic control (*Mex-Gal4/+* and *Hh-IR/+*) flies fed a control diet (CD, gray), high-fat diet (HFD, yellow), and high-sugar diet (HSD, red). n = 20–40 flies per group. Median (midline) and 75% quantiles (dashed lines) are depicted. (B) Sugar PER of *Mex-Gal4>Hh-IR* flies on HFD (yellow line) or respective CD (gray line). n = 39 for each group. (C) HFD-exposed *Mex-Gal4>Hh-IR* flies (filled line) have increased sugar PER compared to genetic control flies (dotted line). n = 20–39 flies. (D) Sugar PER of *Gr64f-Gal4>Hh-IR* flies fed CD (gray line) or HFD (yellow line). (E) HFD does not change *Gr64f-Gal4>Hh-IR* sugar PER compared to genetic control flies fed a HSD. n = 20 flies for each group. (F) Sugar PER of flies expressing *dominant-negative Ptc* (*Ptc*^*1130x*^) in the sweet sensory neurons reverses the effect of HFD compared to CD. n = 39–20 flies. (G) Ptc^1130x^ overexpression does not change sugar PER compared to genetic control flies when fed an HFD. n = 20–39 flies. Data are presented as means ± SEM. PER data were non-normally distributed. Statistical analysis was performed via Mann-Whitney tests (B, D, and F) or Kruskal-Wallis H-tests (A, C, E, and G). *p < 0.05; **p < 0.01; ***p < 0.001; ****p < 0.0001.

Next, we sought to identify the nature of the signal that regulates fat taste perception. Sugar induces the gut to secrete Hh into the circulation where it acts as a signal that suppresses sweet sensation.^15^ To determine whether this secreted Hh suppresses sweet perception while also increasing fat perception, we expressed an *upstream activating sequence* (*UAS*)-driven *Hh inverted repeat* (*Hh-IR*) under the control of the enterocyte-specific driver *Mex-Gal4*. On the low-fat control diet, the hexanoic acid PER of control and knockdown flies was similar (Figure 2A). However, on the HSD, knockdown flies almost lost the fatty acid responsiveness compared to the controls (Figure 2A), which shows that Hh regulates both fatty and sweet taste.

We have previously shown that at sugar levels comparable to the HFD, Hh is released and suppresses sweet taste perception.^15^ Consistently, on the HFD, *Hh* knockdown flies exhibited a pronounced increase in PER, surpassing the response of the control flies (Figures 2B and 2C). On the HFD, *Mex-Gal4>Hh-IR* flies also exhibited increased hexanoic acid PER (Figure 2A), a shift from the requirement shown on HSD. Thus, Hh regulates fatty acid PER, but the direction depends on nutrient state.

To further investigate the role of Hh signaling at HFD, we focused on the local sweet taste neuron Hh signal that the gut Hh regulates. When exposed to HFDs, flies with sweet taste neuron-specific knockdown of *Hh* (*Gr64f-Gal4>Hh-IR*) increased the sugar PER like the control flies (Figure 2G), supporting that there must be a separate fat inductive signal for sweet taste.

To dissect the interplay between the local Hh signal and the potential high-fat signal, we expressed in the sweet taste neurons a dominant-negative version of the Hh receptor Ptc (*Ptc^1130X^*), thereby stimulating the sweet taste neurons’ local Hh signaling pathway and enhancing sugar PER.^15^ Intriguingly, when these flies were transferred to an HFD, it reduced the sugar PER compared to the low-fat control diet (Figures 2F and 2G). Taken together, these findings demonstrate that sugar induces secretion of Hh that regulates both fatty acid and sugar perception and that the consumption of dietary fat triggers the release of a second taste regulatory signal.

### Upd2 from the fat body regulates fat and sweet perception in flies

**Figure 3.**
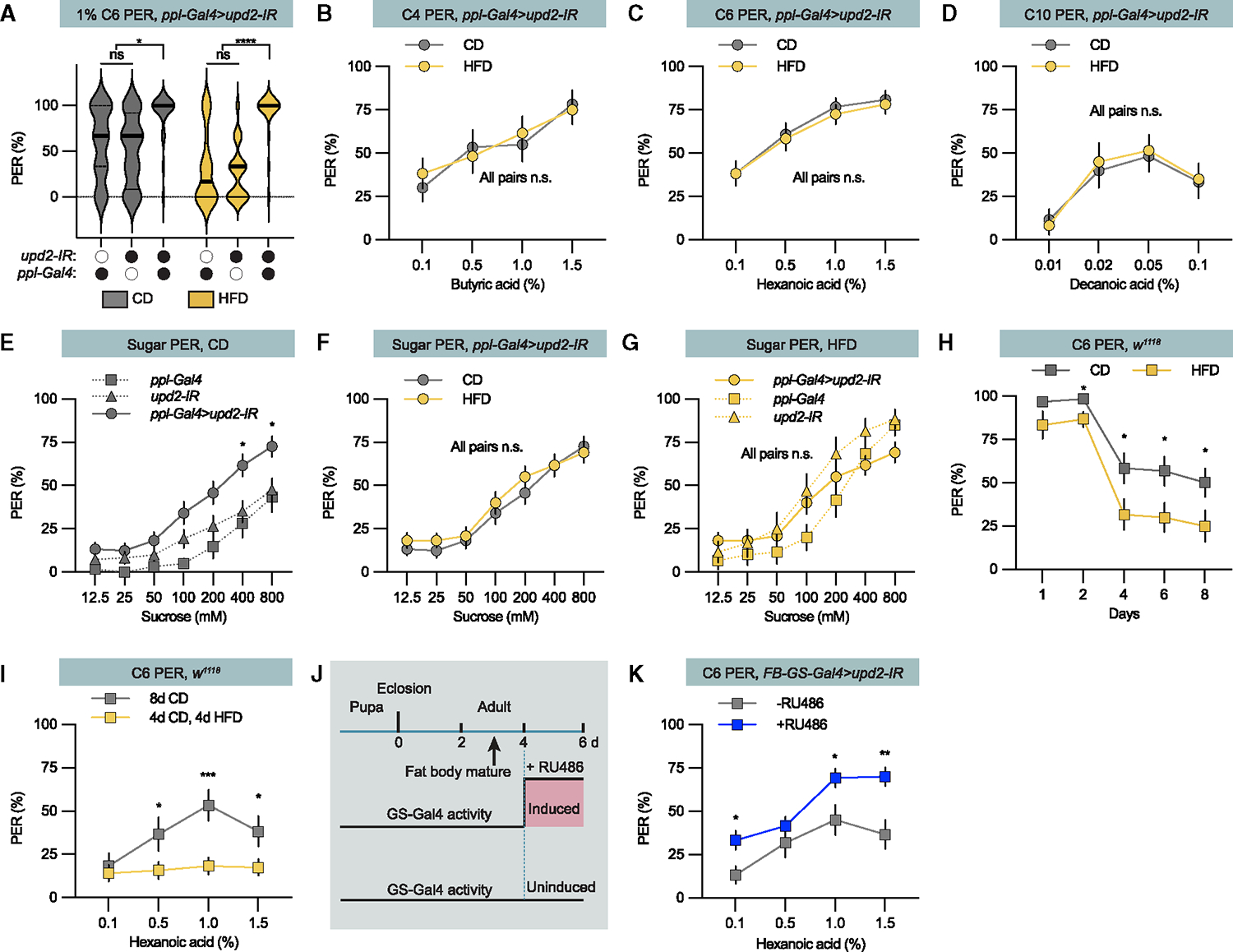
Adipokine Upd2 regulates fatty acid and sugar taste perception (A) 1% C6 PER of *ppl-Gal4>upd2-IR* and genetic control (*ppl-Gal4/*+ and *upd2-IR*/+) flies fed a control diet (CD, gray) or high-fat diet (HFD, yellow). n = 20 for each group. Median (midline) and 75% quantiles (dashed lines) are depicted. (B–D) PER of *ppl-Gal4>upd2-IR* flies fed a high-fat diet (HFD, yellow) and respective control diet (CD, gray line), when stimulated with (B) butyric acid (C4) on butyric acid, (C) hexanoic acid (C6), and (D) decanoic acid (C10). n = 20–40 flies for each group. (E) *ppl-Gal4>upd2-IR* flies on CD show increased sugar PER compared to genetic control flies. n = 20–40 flies per group. (F) *ppl-Gal4>upd2-IR* flies on CD (gray line) and HFD (yellow line) show similar sugar PER. n = 20–40 flies. (G) HFD-fed *ppl-Gal4>upd2-IR* and control flies exhibit a similar sugar PER. n = 20–40 flies. (H) Hexanoic acid PER dynamics during the first 8 days for flies on CD (gray) and HFD (yellow). n = 20 for each group. (I) Hexanoic acid PER after CD for 8 days or 4 days on CD and 4 days on HFD. n = 20 for each group. (J) Schematic illustration of the GeneSwitch experiments. Adult fat body matures around day 3. Presence of the drug RU486 induce FB-GS-Gal4 activity and expression. (K) Hexanoic acid PER of *FB-GS-Gal4>upd2-IR* on control diet with +RU486 (blue line) or without –RU486 (gray line). n = 20 for each group. Data are presented as means ± SEM. PER data were non-normally distributed. Statistical analysis was performed via Mann-Whitney tests (B–D, F, H, I, and K) or Kruskal-Wallis H tests (A, E, and G). *p < 0.05; **p < 0.01, and ***p < 0.001. For more information, please see Figures S1–S3.

We hypothesized that the signal triggered by excess fat intake was Upd2, which the fat body releases in proportion to fat stores.^24,25^ To determine whether Upd2 regulates fatty acid taste perception, we knocked down *Upd2* in the fat body by expressing *UAS-Upd2* inverted repeat (*Upd2-IR*) under the control of the fat-body-specific *ppl-Gal4* driver. Irrespective of dietary conditions, the fat body *Upd2* knockdown flies increased the hexanoic acid PER to almost 100% responders (Figure 3A). This shows that level of Upd2 suppression of the fatty acid taste set the response to hexanoic acid, even under low-fat conditions. We further expanded the study to include the short and long fatty acids and showed that the knockdown flies lost the high-fat suppression of fatty acid taste (Figures 3B–3D). Thus, the level of Upd2 signaling set the fatty acid taste perception level.

Next, we asked whether Upd2 also regulates sweet taste perception. In alignment with our findings regarding fatty acid PER, the *Upd2* fat body knockdown flies displayed consistent and increased sugar PER level irrespective of experimental diet (Figures 3E–3G and S2). This PER level was similar to the one of control flies exposed to HFD (Figure 3), indicating that the degree of suppression from Upd2 signaling determines the sweet taste perception. Together our results show that Upd2 signaling from the fat body suppresses both fat and sweet taste perception.

### Dietary fat and Upd2 regulate fatty acid taste independent of fat body maturity

The adult fat body matures 3–4 days after eclosion.^7,32^ To analyze if the fatty acid taste matures, we determined control flies’ hexanoic acid PER for 8 days (Figure 3H). The hexanoic acid PER transitioned from high levels during the first 2 days after eclosion to a lower adult baseline, suggesting that the transition might be linked to fat body development. Flies on the HFD showed a similar drop in hexanoic acid PER to an adult baseline. The HFD suppressed the hexanoic acid PER directly before the drop and produced a lower baseline compared to the flies on low-fat control diet (Figure 3H), indicating that HFD suppresses the fatty acid PER independent of fat body development. We further tested this by postponing exposure to the HFD from directly after eclosion to day 4. This delay in exposure also resulted in hexanoic acid PER suppression (Figure 3I), showing that the suppression is parallel to the fat body development.

To explore whether Upd2’s suppression of fat taste correlated with fat body maturation, we used the *fat body gene switch Gal4* (*FB-Gs-Gal4, 106*^33^) to induce the expression of the *Upd2 inverted repeat* from day 4 post eclosion. This refined Gal4/UAS mechanism initiates transgene expression in *Drosophila* when the drug RU486 is administered (Figures 3J, S3A, and S3B). When *FB-GS-Gal4; Upd2-IR* flies on control diet were exposed to RU486 after day 4, the fatty acid PER increased compared to the uninduced flies (Figure 3K, S3F, and S3G). The *FB-GS-Gal4* control flies exposed to RU486 showed no increase (Figure S3). We also overexpressed Upd2:GFP with *FB-GS-Gal4*, and administration of RU486 at day 4 induced suppression of the different fatty acids’ PER (Figures S3H–S3J). Thus, Upd2 is both necessary and sufficient to suppress fatty acid taste perception.

### Fat overconsumption induces fat body Upd2 expression and secretion

**Figure 4.**
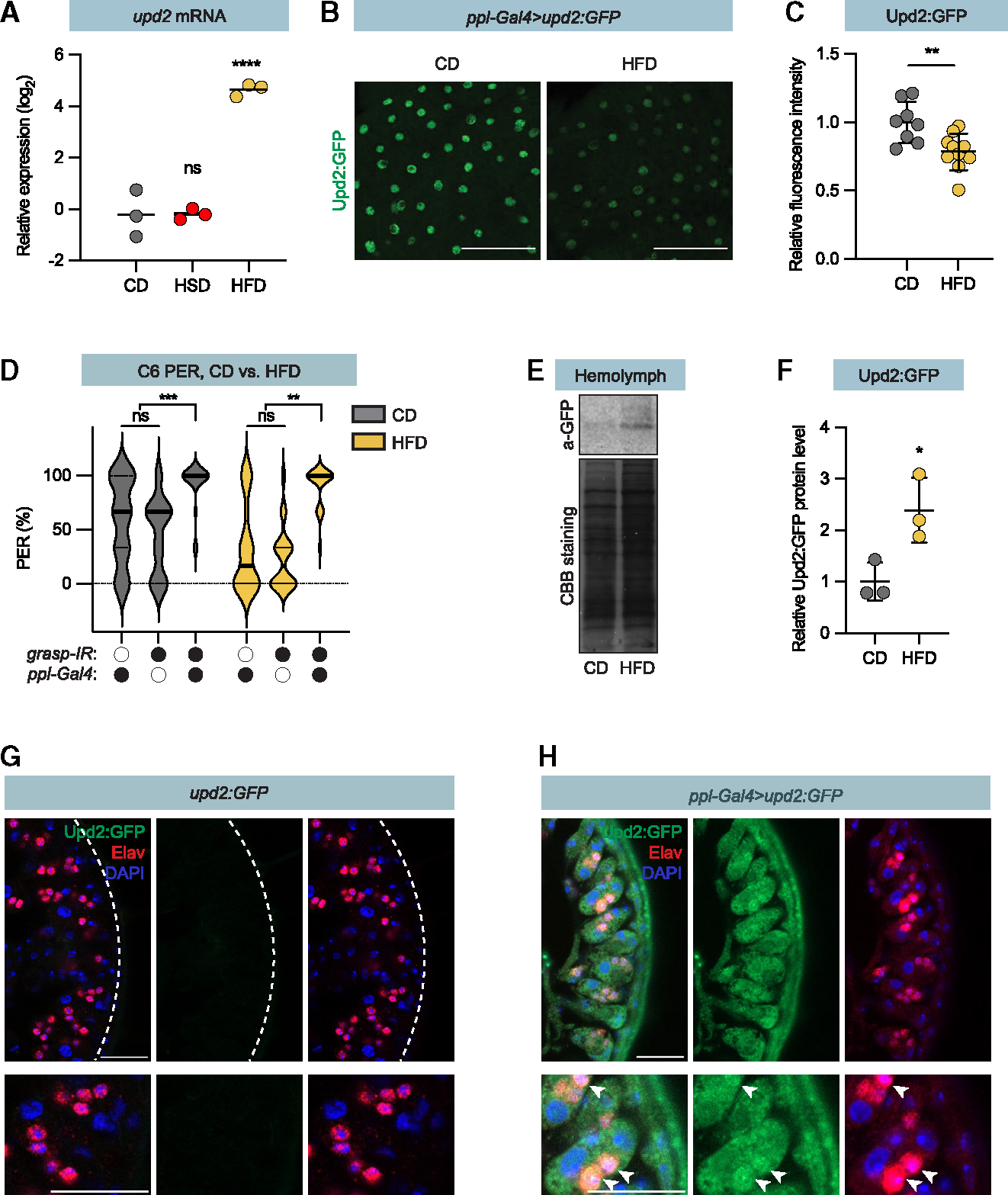
Fat body secretion of Upd2 to hemolymph and uptake in taste sensory neurons (A) qRT-PCR analysis of *Upd2* fat body expression in 4-day-old adult flies shifted to the indicated diets at eclosion. Control diet (CD), high-sugar diet (HSD), and high-fat diet (HFD). Middle lines depict average. (B) Representative confocal images of adult fat body (*ppl-Gal4>Upd2:GFP*). Adult flies were fed the CD (left) or HFD (right) for 4 days. Upd2:GFP appears in green. Scale bars, 50 μm (C) A plot quantifying the relative Upd2:GFP fluorescence intensity of the experiments in (B). (D) Hexanoic acid PER of *ppl-Gal4>grasp-IR* and genetic control flies on CD (gray) or HFD (yellow). Median (middle line) and 75% quantiles (dashed lines) are shown. n = 20 flies (E) Upper panel, anti-GFP western blot of total protein from adult hemolymph of *ppl-Gal4>Upd2:GFP* flies on CD or HFD; lower panel, Coomassie brilliant blue (CBB) staining of the membrane. (F) Quantification of relative Upd2:GFP protein levels in the hemolymph. n = 3. Data are presented as means ± SD. Middle lines depict average. (G and H) Representative images of the labellum in *upd2:GFP* (G) and *ppl-Gal4>upd2:GFP* (H) flies. Upd2:GFP appears in green, anti-Elav (neuron nuclei) is shown in red, and DAPI (DNA) appears in blue. Scale bars, 20 mm. Data are presented as means ± SD (C and F) or means ± SEM (D). *p < 0.05; **p < 0.01; ***p < 0.001; ****p < 0.0001. Statistical analysis was performed using the one-way ANOVA with Dunnett’s correction (A), t tests with Welch’s correction (C and F), or Mann-Whitney tests (D).

To explore the relationship between *Upd2* expression in the fat body with sugar and fat consumption, we placed flies on different diets and examined expression changes via qPCR. After 5 days on the HFD, Upd2 mRNA levels in male flies increased several folds compared to those raised on the low-fat control diet (Figure 4A). In contrast, flies on the isocaloric HSD (34%, 1 M sucrose) did not exhibit higher *Upd2* expression (Figure 4A). This confirmed that fat ingestion drives *Upd2* expression.^24^

To determine whether the HFD induces Upd2 secretion, we expressed *UAS-Upd2:GFP* under the control of *ppl-Gal4* and visualized the resulting GFP expression. In flies raised on the low-fat diet control diet, Upd2:GFP remained localized in the fat cells in large punctate structures (Figure 4B), consistent with previous descriptions of lipid droplet accumulation.^25^ This signal, however, was reduced in flies exposed to an HFD (Figures 4B and 4C), suggesting that secretion of Upd2:GFP is in response to fat ingestion.

Upd2 is secreted in an unconventional manner, bypassing the Golgi in steps controlled by *Grasp65*.^25^ Consequently, *Grasp65* knockdown also phenocopied *Upd2* knockdown and increased hexanoic acid perception in flies exposed to either HFD or low-fat control diet (Figure 4D). We further quantified Upd2:GFP in the hemolymph via western blot. Consistent with the hypothesis that fat ingestion induces Upd2 secretion, we found increased Upd2:GFP hemolymph levels in flies exposed to the HFD (Figures 4E and 4F). Together, these results support that fat intake regulates fat body Grasp65 function and Upd2 secretion.

### Secreted Upd2 is stored in taste neurons

To identify possible target cells for the secreted Upd2, we visualized across the body the localization of fat body secreted Upd2:GFP. We observed that Upd2:GFP localized to the labellum and taste neurons (Figures 4G and 4H). Interestingly, the Upd2:GFP was taken up and localized to the nucleus of taste neurons (Figure 4H). Recently the nucleus was also identified as an Upd2 reservoir in other tissues,^34^ suggesting that Upd2 is taken up and stored in taste neurons. Interestingly, Hh secreted from the gut is stored in taste sensilla lymph.^15^ Thus, both Upd2 and Hh are taken up from the hemolymph and stored in sensory tissues, suggesting that the local level of the ligands is higher than in the circulation. These results further suggest Upd2 connects fat uptake in the fat body with the function of taste neurons.

### HFD and Upd2 induce JAK/STAT signaling in sweet taste neurons

**Figure 5.**
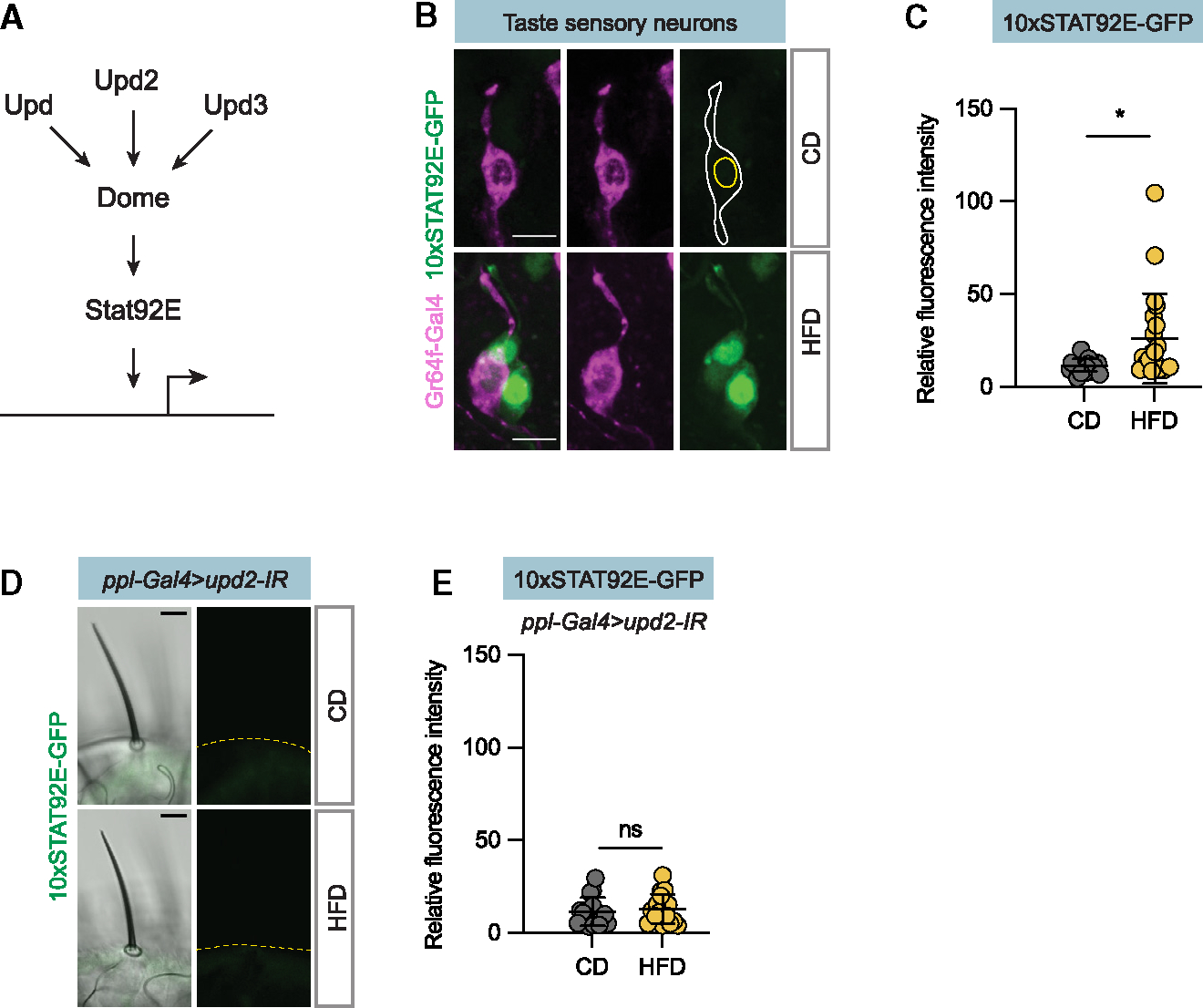
Fat-body-derived Upd2 regulates JAK/STAT signaling in sweet sensory neurons (A) A schematic of the JAK/STAT signaling pathway. Dome is activated by three ligands: Upd1, Upd2, and Upd3. Stat92E is a downstream transcription factor. (B) Confocal images of the adult labellum from *10xSTAT92E-GFP* flies fed CD and HFD. Gr64f sweet sensory neurons appear in magenta, and GFP appears in green. (C) Relative fluorescence intensity of 10x STAT92E-GFP from the experiments in (B); n = 24 and 21, respectively. (D) Representative confocal images of taste sensilla of *ppl-Gal4>Upd-IR; 10xTAT92E-GFP* flies on CD and HFD; GFP appears in green. (E) Relative fluorescence intensity of labella of *ppl-Gal4>Upd-IR; 10xTAT92E-GFP* on CD and respective HFD. Data represent mean ± SD. Scale bars, 5 μm. Statistical analysis was performed using two-tailed Mann-Whitney tests. *p < 0.05.

To ask whether dietary fat regulates JAK/STAT signaling (Figure 5A) in taste neurons, we used a JAK/STAT reporter, *10xStat92E-GFP*. This reporter is induced by Stat92E protein and requires JAK/STAT signaling for expression.^35^ In flies raised on the low-fat control diet, we barely detected any *10xStat92E-GFP reporter* expression in sweet taste neurons (Figures 5B and 5C). However, exposure to HFD increased *10xStat92E-GFP* reporter expression in the sweet taste neurons and other taste neurons (Figures 5B and 5C), indicating that dietary fat induces JAK/STAT signaling in the sweet taste neurons.

Upd2 is one of three Upd ligands that activates the JAK/STAT signaling pathway (Figure 5A). Thus, to determine if the induced reporter expression is downstream of fat-body-derived Upd2, we subjected *ppl-Gal4>Upd2-IR, 10xStat92E-GFP* flies to an HFD. Strikingly, these flies failed to exhibit the increase in marker expression seen in the control group (Figures 5D and 5E). Accordingly, these experiments together show that Upd2, secreted from the fat body in response to dietary fat, initiates the JAK/STAT signaling cascade within sweet taste neurons.

### Dome and Stat92E regulate sweet taste neuron-mediated taste perception

**Figure 6.**
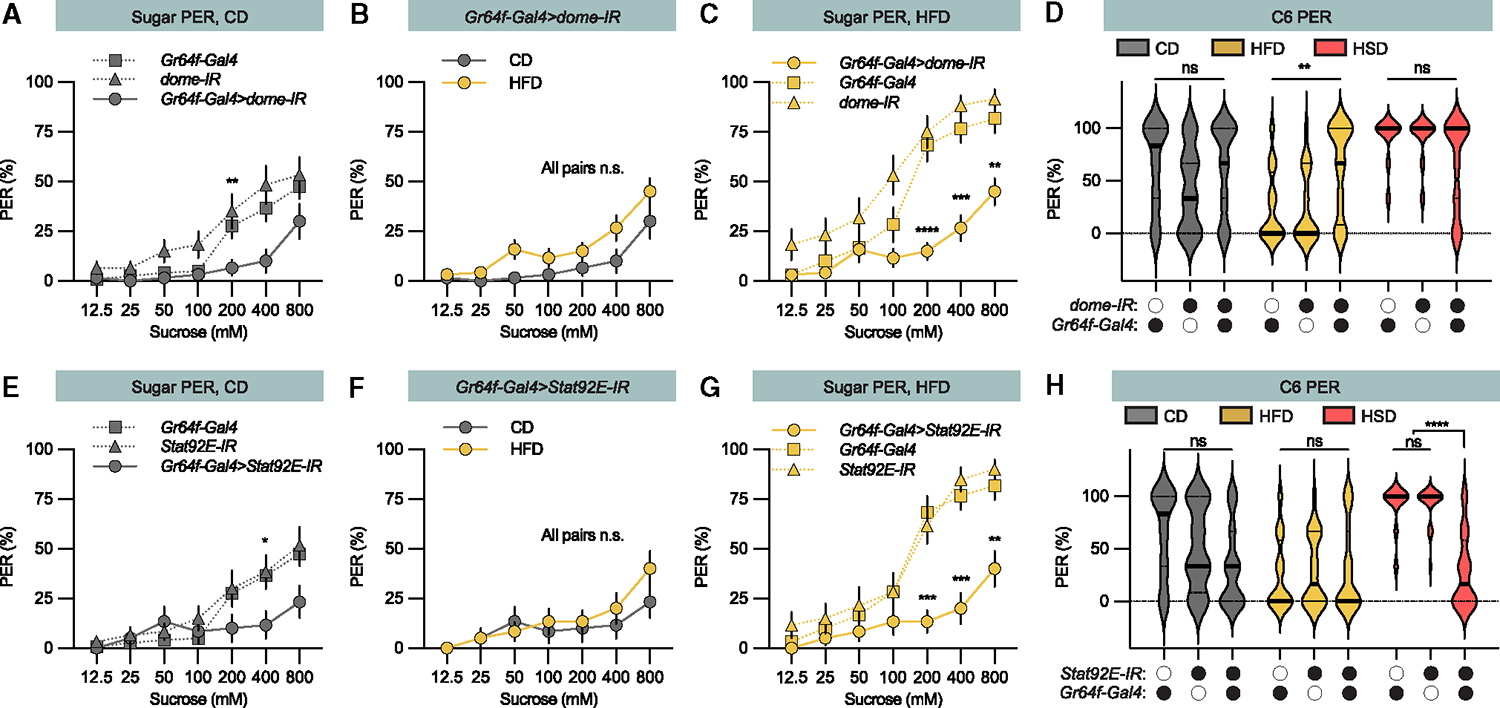
The JAK/STAT pathway mediates the effect of dietary fat on sweet sensation and fatty acid perception (A) *Gr64f-Gal4>dome-IR* flies on control diet (CD) show decreased sugar PER (filled line) compared to genetic control flies (*Gr64f-Gal4/+ and dome-IR*/+ dotted lines). n = 20–40 flies per group. (B) *Gr64f-Gal4>dome-IR* flies on CD (gray line) and HFD (yellow line) show similar sugar PER. n = 20–40 flies (C) HFD-fed *Gr64f-Gal4>dome-IR* flies show lower sugar PER compared to the genetic controls (*Gr64f-Gal4/+ and dome-IR/+)*; n = 20–40 flies. (D) Hexanoic acid PER of *Gr64f-Gal4>dome-IR* and genetic control (*Gr64f-Gal4/+ and dome-IR/+*) flies fed CD (gray), HFD, (yellow), and HSD (red); n = 20–40 flies. (E) *Gr64f-Gal4>Stat92E-IR* flies on CD show decreased sugar PER (filled line) compared to genetic control flies (*Gr64f-Gal4/+ and Stat92E-IR*/+ dotted lines). (F) *Gr64f-Gal4>Stat92E-IR* flies on CD (gray line) and HFD (yellow line) show similar sugar PER. n = 20. (G) HFD-fed *Gr64f-Gal4>Stat92E-IR* show lower sugar PER compared to the genetic controls (*Gr64f-Gal4/+ and Stat92E-IR/+*); n = 20. (H) Hexanoic acid PER of *Gr64f-Gal4>Stat92E-IR* and genetic control (*Gr64f-Gal4/+ and Stat92E-IR/+*) flies fed CD (gray), HFD (yellow), and HSD (red); n = 20–40 flies. Data are presented as means ± SEM. Median (midline) and 75% quantiles (dashed lines) are depicted (D and H). Statistical analysis was performed with two-tailed Mann-Whitney tests (B and F) or Kruskal-Wallis H tests (A, C–E, G, and H). *p < 0.05; **p < 0.01; ***p < 0.001; ****p < 0.0001. For more information, please see Figure S4.

Having shown that Upd2 initiates JAK/STAT signaling in sweet taste neurons, our focus shifted to investigating how the JAK/STAT pathway regulates the sweet taste neurons and taste preferences. First, we knocked down the Upd receptor *Domeless* (*Dome*) in sweet taste neurons using *Gr64f-Gal4*. The Dome knockdown flies exhibited reduced sugar PER in comparison to the control group (Figure 6A) and lost the increase in sweet perception after exposure to an HFD (Figures 6B and 6C). Furthermore, the Dome knockdown flies displayed a reduced suppression of hexanoic acid PER when subjected to the HFD (Figure 6D). Thus, Upd2 through Dome regulates both sweet and fatty acid taste perception.

Next, we knocked down the Upd-regulated transcriptional activator *Stat92E* in sweet taste neurons. Similar to the *Dome* knockdown flies, the *Gr64f-Gal4>Stat92E-IR* flies showed a reduced sugar PER compared to control flies (Figure 6E). These knockdown flies also lacked the HFD-induced increase in sweet perception (Figures 6F and 6G), again confirming that Upd2 mediates the effect of fat and regulates the sweet taste neurons’ JAK/STAT pathway that controls sugar PER. In contrast to the *Dome* knockdown that increased the hexanoic acid PER, the *Stat92E* knockdown showed a reduced fatty acid PER, regardless of the diet (Figure 6H), suggesting that Stat92E is required for sweet taste and to counter act Dome and the suppression of the fatty acid taste.

### Negative feedback within the JAK/STAT pathway determines fatty acid perception

**Figure 7.**
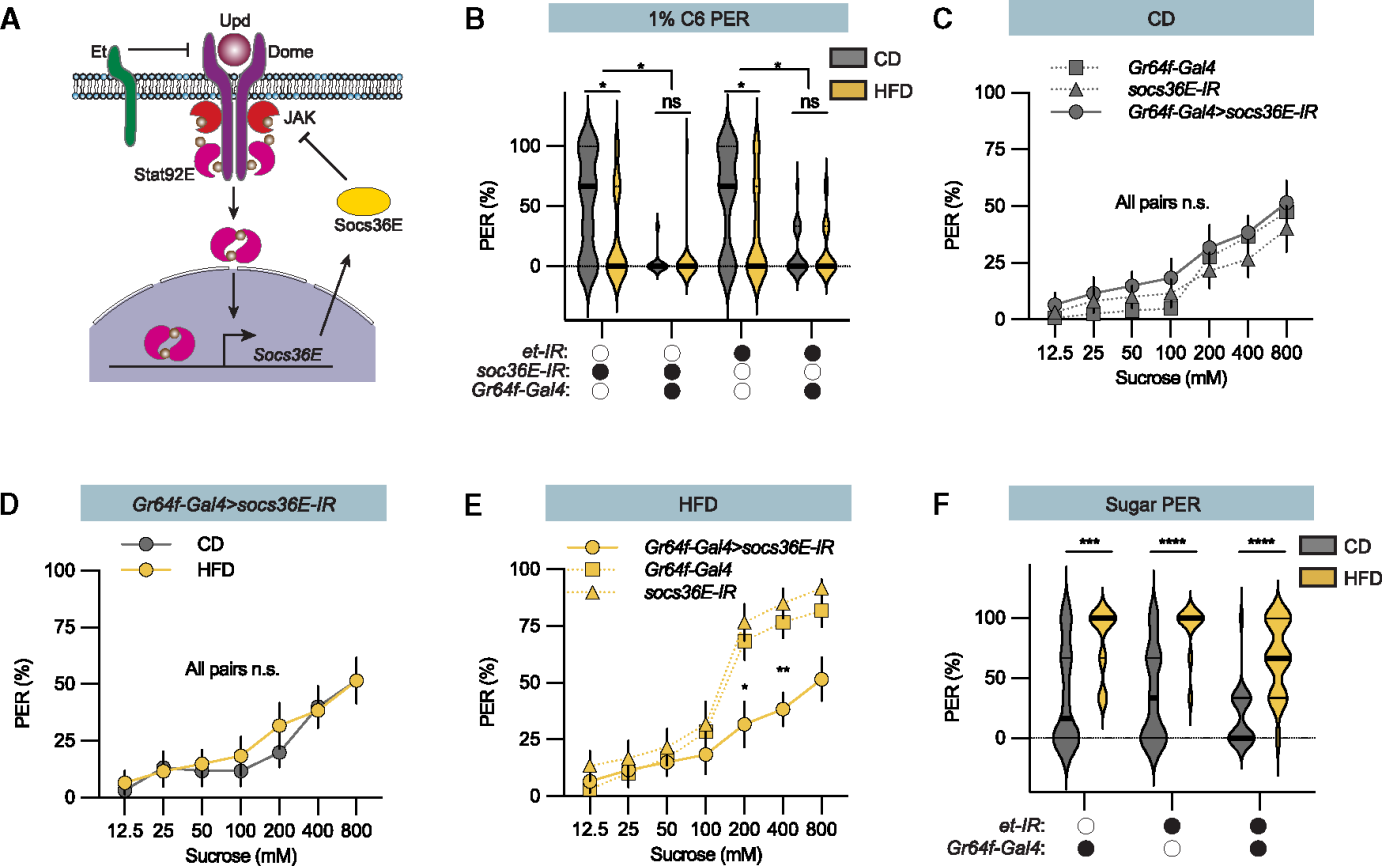
Socs36E and Et regulate sweet and fatty acid perception (A) Schematic of the JAK/STAT signaling pathway. Both the short receptor Et and the downstream JAK/STAT mediator Socs36E act as negative regulators of the JAK/STAT pathway. (B) 1% C6 PER of *Gr64f-Gal4>et-IR* and *Gr64f-Gal4>Socs36E-IR* flies compared to *et-IR/+* and *Socs36E-IR*/+ flies, fed control diet (CD, gray) and high-fat diet (HFD, yellow). n = 20. Median (midline) and 75% quantiles (dashed lines) are depicted. (C) *Gr64f-Gal4>Socs36E-IR* flies on CD show decreased sugar PER (filled line) compared to genetic control flies (*Gr64f-Gal4/+ and socs36E-IR*/+, dotted lines). n = 20. (D) *Gr64f-Gal4>Socs36E-IR* flies on CD (gray line) and HFD (yellow line) show similar sugar PER. n = 20. (E) HFD-fed *Gr64f-Gal4>Socs36E-IR* show lower sugar PER compared to the genetic controls (*Gr64f-Gal4/+ and Socs36E-IR*/+, dotted lines); n = 20. (F) Sugar PER of *Gr64f-Gal4>et-IR* and genetic control (*Gr64f-Gal4* and *et-IR/+*) flies exposed to CD (gray) and HFD (yellow). n = 20. Median (midline) and 75% quantiles (dashed lines) are depicted. Data are presented as means ± SEM. PER data were non-normally distributed. Statistical analysis was performed via Mann-Whitney tests (B, D, and F) or Kruskal-Wallis H tests (C and E). *p < 0.05; **p < 0.01; ***p < 0.001; ****p < 0.0001.

Our results this far suggest that negative feedback from Stat92E possibly balances the fat and Upd2 induced fatty acid suppression. The JAK/STAT pathway has several inbuilt negative feedback systems that target Dome function (Figure 7A). During development, Stat92E induces the expression of *Suppressor of cytokine signaling 36E* (*Socs36E*) that promotes Dome degradation.^36–38^ Flies with knockdown of *Socs36E* in sweet taste neurons almost lacked hexanoic acid PER (Figure 7B), consistent with the hypothesis that Socs36E negative feedback determines fatty acid perception. Another negative JAK/STAT regulator, Et, inhibits JAK/STAT signaling when it forms a heterodimeric complex with Dome.^36,39,40^ We found knockdown of *Et* in sweet taste neurons also suppressed the fatty acid PER (Figure 7B), suggesting that any negative regulation of Dome increase fatty acid perception.

Despite the strong effect loss of the negative feedback has on fatty acid perception across diets, *Socs36E* knockdown flies showed a more modest effect on sweet perception (Figure 7C). The knockdown flies showed increased sweet perception on the HFD, but their sugar PER on the balanced low-fat control diet was like that of control flies (Figures 7C–7E). Consistent with this reduced effect, knockdown of *Et* had no significant effect on sugar perception (Figure 7F). Thus, it is possible that Hh buffers changes in sweet perception on the balanced low-fat control diet. Nevertheless, our results show that Upd2 exerts its effect on taste perception via two signaling pathways downstream of Dome. A non-canonical Dome signal suppresses fatty acid taste perception, and the JAK/STAT signal regulates sweet sensation and governs fatty acid perception via negative feedback on Dome.

## DISCUSSION

In this study, we investigate how sugar and fat overconsumption regulate taste perception in *Drosophila melanogaster*. Consistent with our previous work, we find that gut-derived Hh serves as a proxy for sugar.^15^ In *Drosophila*, fat and sugar activate sweet taste cells, and our expectation was that this signal would suppress both sugar and fatty acid taste perception and thus regulate caloric intake. However, we demonstrate that the sugar-induced Hh signal increases fatty acid taste sensitivity and suggests a nutrient-balancing effect. Our results also show that the gut Hh signal shifts from enhancing to suppressing fatty acid taste perception under an HFD. Thus, sugar and the gut-released Hh regulate taste to balance fat and sugar nutrient intake and not caloric intake.

Our diet experiments further demonstrate a reciprocal relationship between the two macronutrients in which fat consumption increases sweet sensation and sugar consumption enhances fat taste perception. We also demonstrate that fat overconsumption suppresses fatty acid perception much like sugar overconsumption suppressed sweet taste. Our results demonstrate that Upd2 is the fat taste suppressive signal. We show that fat overconsumption induces fat body expression and secretion of Upd2 that activates JAK/STAT signaling in the sweet taste neurons. Our mechanistic study further shows that fatty acid taste suppression is directly downstream of Dome, whereas the sweet taste regulation requires JAK/STAT signaling. Together these results demonstrate that there are two proxy signals, Hh for sugar and Upd2 for fat, that both regulate fatty acid and sweet taste.

Our results show that Upd2, similar to Hh,^15^ is taken up and stored by the taste neurons. For both ligands, the reservoir increases the ligand’s local concentration. The increase in ligand concentration is likely vital because most signaling molecules must meet a specific threshold concentration for effectiveness. The gut secretion of Hh during development also does not affect wing development,^41^ which together with our data suggests that the endocrine signals may require local accumulation to achieve effective levels. Another reason for the reservoir is likely to reduce fluctuations in ligand and taste regulation. Accumulation of the ligand likely takes time, and uptake can be regulated, and the reservoir levels do not necessarily reflect the hemolymph levels. Thus, the reservoir increases ligand levels and at the same time makes ligand levels and the taste signal robust to quick metabolic changes.

Our mechanistic analysis further shows that the fatty acid taste perception level is regulated by negative feedback on Dome. We find that two negative factors in the Upd2 pathway, Et and Socs36E, dramatically affect fatty acid perception. The two have different effects on JAK/STAT signaling, with Et sequestering Dome in signaling-incompetent complexes^42^ and Socs36E inhibiting both Dome and STAT activation.^38,43^ Et also only regulates fatty acid perception, whereas Socs36E regulates both fat and sugar taste. Simple negative feedback systems, like the Dome-Stat92E-Socs36E-Dome, tend to slow down regulation and make it more robust to changes. Thus, the storage and feedback loops of Hh and Upd2 suggest that many cycles of consumption and taste are continuously interacting to form a sort of ‘‘memory’’ of the sugar-respective fat state. This we also observe in flies raised on the balanced low-fat control diet. In this condition, although Upd2:GFP is only found at low levels in the hemolymph, Socs36E is still required to avoid loss of fatty acid taste. This suggests that Upd2 levels are not limiting and cause a sufficient activation of Dome even on balanced diets. It also suggests that Upd2 and Hh, rather than acting as a gradual signal, communicate the presence of fat and sugar in the diets.

Collectively, our findings underscore taste sensitivity as a product of both immediate feeding experience and cumulative nutritional history. We identify that the taste regulatory mechanisms between sugar and fat have similarities, in that they are slow and act over several meals, functioning as a body memory of the feeding and nutritional status. The signals form a closed regulatory loop in which the final component, the nutrient level in the body, feeds back on the start, taste and food identification. Thus, this body-taste axis when in balance probably sets taste perception levels that best support the metabolic needs of the animal. Consequently, if intake becomes dysregulated, it might reach a point where the taste regulatory mechanisms described here can speed up rather than inhibit sugar and fat malnutrition and advance to obesity and metabolic disease.

### Limitations of the study

(1) We have only studied males; how females and the changes in metabolism after mating are linked to Upd2 and Hh remain for investigation. (2) We have not studied the secretion of the endogenous Upd2. Thus, we cannot show the full magnitude of the Upd2 uptake and dynamics. (3) We have not studied how Upd2 regulates food intake or foraging strategies. Feeding and free running behavior studies are needed for such conclusions. (4) We have only investigated fat and sweet taste sensation. It seems likely that micronutrients (vitamins, salts, and minerals) also have dedicated signals that would be interesting to identify.

## STAR★METHODS

### RESOURCE AVAILABILITY

#### Lead contact

Further information and requests for resources and reagents should be directed to and will be fulfilled by the lead contact, Mattias Alenius (mattias.alenius@umu.se).

#### Materials availability

This study did not generate new unique reagents.

#### Data and code availability

- All data reported in this paper will be shared by the lead contact upon request.
- This paper does not report original code.
- Any additional information required to reanalyze the data reported in this paper is available from the lead contact upon request.

### EXPERIMENTAL MODEL DETAILS

#### *Drosophila* strains and husbandry

The following *Drosophila* strains were used for tissue-specific transgene expression: *ppl-Gal4* (Bloomington *Drosophila* Stock Center, BDSC_58768) and *FB-GS-Gal4* (BDSC_8151) for the fat body and *Gr64f-Gal4* (BDSC_57669) for sweet taste neurons. The following RNAi lines were employed for manipulation of JAK/STAT signaling: *Upd2-IR* (BDSC_33988), *dome-IR* (Vienna *Drosophila* Resource Center, VDRC_106071), *Stat92E-IR* (VDRC_43867), *et-IR* (BDSC_42557), and *socs36E-IR* (BDSC_35036).

For general handling, 50–100 virgin females were crossed with 10–20 males and maintained in bottles on the 6% control diet. All flies were reared in a 25°C incubator on a 12h dark/12h light cycle under constant 60% humidity, unless otherwise mentioned. The parental flies were flipped into new bottles or disposed of after 2–3 days. Within 12 h of eclosion, the flies were collected and transferred, dependent on experiment, into fresh 6% or 34% sugar food vials or 14% high-fat diet vials (22–25 flies/vial). The recipe for each type of food appears in Table S1.

### METHOD DETAILS

#### Hemolymph analysis

Hemolymph was collected from 4-day-old adult males (*ppl-Gal4>Upd2:GFP*) exposed to the indicated diets. A small opening was cut in the abdomen of each fly and they were placed in a perforated 0.5-mL tube (20 flies/tube) inserted into a 1.5-mL tube containing 5 μL of 2x SDS loading buffer. Hemolymph was then collected via centrifugation (10,000 rpm for min). Total protein was separated on 10% Bis-Tris Protein Gels and transferred onto PVDF membranes (pore size 0.45 μm, Immobilon-P, Thermo Scientific) under a constant current of 200 mA for 90 min. The membranes were blocked with 5% milk in 1x TBST for 2 h at room temperature. Incubation with the primary antibody, mouse anti-GFP (1:5000, mab3580, Millipore) was performed on a rotating shaker at 4°C overnight. Then, the blots were washed and incubated with the secondary antibody HRP conjugated anti-mouse (1:5000, #7076, Cell Signaling). The membranes were developed with the Azure 600 imaging system. The protein levels were quantified using Fiji software and normalized against Coomassie Brilliant Blue (CBB) stained total protein on the membranes.

#### Behavior assays

Proboscis Extension Response (PER) assays were performed using a protocol modified from.^44^ The behavior assays were carried out at zeitgeber time ZT3–6. The flies were anesthetized on ice, mounted into 200-μL pipette tips (Cat# 89079–476, VWR) cut so that only the fly’s head was exposed, and aligned on a glass slide using double-sided tape. The flies were placed in a humid chamber and allowed to recover for 60–90 min. Before the assay, the flies were stimulated with water and allowed to drink until satiated. Tastants were then introduced using a 200-μL pipette tip attached to a 1-mL syringe. For the sucrose PER, each stimulation was less than a second. For the fatty acid PER, hexanoic acid was applied for up to 5 s. Flies were allowed to drink water in between tests. The *Drosophila* labellum was stimulated three times for each tastant with a 1 min intertrial interval. The fly that showed full proboscis extension was recorded as 1, otherwise was recorded as 0. Thus, for each fly, the total number PER would be 0, 1, 2, or 3, and calculated as a percentage of response 0%, 33.3%, 66.7%, and 100%, respectively.

#### Quantitative PCR

To quantify diet induced changes in fatbody Upd2 expression, total RNA was extracted from the adult abdomen (sans intestines and reproductive organs) from 25 to 30 adult male flies with RNeasy Mini Kit (74104, Qiagen, USA). Three to seven bioreplicates was performed per data point. The samples were directly stored on ice in RNAlater (Qiagen). The RNA was transcribed into cDNA using the iScript cDNA Synthesis Kit (1708890, Bio-Rad, USA). Quantitative PCR was performed using the iTaq Universal SYBR Green Supermix (1725121, Bio-Rad, USA) in a Bio-Rad CFX Connect Real-Time PCR Detection System. We corrected the expression of all samples to that of actin and used an average of the corrected control sample from the experiment as reference. Relative expression was determined as 2^−ΔΔCT^. Statistical significance was assessed using Student’s t-tests and ANOVAs with corrections for multiple comparisons.

#### Immunohistochemistry

Newly eclosed adult flies were collected into fresh food vials (22–25 flies/vial) and placed on the indicated diets. Four days after collection, the proboscises or guts were dissected and fixed in 4% PFA for 30 min at room temperature. The primary antibodies were mouse monoclonal anti-Elav 1:100 (9F8A9, Developmental Studies Hybridoma Bank, DSHB) and chicken anti GFP 1:1000 (Abcam ab13970). The secondary antibodies were donkey anti-mouse IgG (H + L) Alexa Fluor 647 1:500 (Cat# 715–605-151, Jackson ImmunoResearch) donkey anti-chicken IgY (H + L) Alexa Fluor 488 1:500 (Cat# 703–545-155, Jackson ImmunoResearch). Confocal microscopy images were collected on either a Leica SP8 platform or a Zeiss LSM900 confocal. Each experiment was repeated at least three times and images processed from 6 animals. Relative fluorescence intensity in each confocal image was quantified using the Fiji software (https://imagej.net/Fiji).

### QUANTIFICATION AND STATISTICAL ANALYSIS

#### Data analysis

Relative fluorescence intensity was quantified from raw confocal images using the Fiji software (https://imagej.net/Fiji). Statistical analyses and data plotting were performed using GraphPad Prism 9. Data normality was tested via the Shapiro-Wilk test. Normally distributed data were analyzed via two-tailed t-tests with Welch’s correction or a one-way ANOVA followed by Tukey’s correction. Since the behavioral data were non-normally distributed, they were analyzed via either two-tailed Mann-Whitney test (for two groups) with Bonferroni’s correction or Kruskal-Wallis H-tests with Dunn’s corrections for multiple comparisons. Asterisks denote statistical significance. Boxplots show the median and the first and third quartile, with whiskers indicating the full range of values. Violin plots show the median (midline) and 75% quantiles. No data were excluded. Sample-size calculations were not performed. Instead, sample size was chosen on the basis of similar previously published studies of *Drosophila* behavior and metabolism.^45,46^ Figures were generated using Adobe Illustrator.

## Supplementary Material

Supplemental

## Figures and Tables

**KEY RESOURCES TABLE:** 

REAGENT or RESOURCE	SOURCE	IDENTIFIER

Antibodies		

Mouse monoclonal anti-Elav	Developmental studies Hybridoma bank (DSHB);	Cat# Elav-9F8A9; RRID:AB_528217
Rabbit polyclonal anti-GFP	Abcam	Cat# ab290; RRID:AB_303395
Donkey anti-mouse IgG (H + L) Alexa Fluor^®^ 647	Jackson ImmunoResearch	Cat# 715-605-151; RRID:AB_2340863
Goat anti-Rabbit IgG (H + L) Secondary Antibody, HRP	Thermo Fisher Scientific	Cat# 31460; RRID:AB_228341

Chemicals, Peptides, and Recombinant Proteins		

Butyric Acid	MilliporeSigma	Cas# 107-92-6
Hexanoic acid	MilliporeSigma	Cas# 142-62-1
Decanoic Acid	MilliporeSigma	Cas# 334-48-5
Mifepristone (RU486)	MedChemExpress	Cas# 84371-65-3

Experimental Models: Organisms/Strains		

*Drosophila melanogaster. w* ^*1118*^	Bloomington Drosophila Stock Center (BDSC)	RRID: BDSC_3605
*Drosophila melanogaster. UAS-Hh-IR*	BDSC	RRID: BDSC_32489
*Drosophila melanogaster. UAS-ptc* ^*1130X*^	BDSC	RRID: BDSC_52215
*Drosophila melanogaster. Gr64f-Gal4*	BDSC	RRID: BDSC_57669
*Drosophila melanogaster. Mex-Gal4*	BDSC	RRID: BDSC_91368
*Drosophila melanogaster. UAS-GFP.nls*	BDSC	RRID: BDSC_4776
*Drosophila melanogaster. ppl-Gal4*	BDSC	RRID: BDSC_58768
*Drosophila melanogaster. upd2-IR*	BDSC	RRID: BDSC_33988
*Drosophila melanogaster. et-IR*	BDSC	RRID: BDSC_42557
*Drosophila melanogaster. socs36E-IR*	BDSC	RRID: BDSC_35036
*Drosophila melanogaster. FB-GS-Gal4*	BDSC	RRID: BDSC_8151
*Drosophila melanogaster. dome-IR*	Vienna *Drosophila* Resource Center (VDRC)	VDRC_106071
*Drosophila melanogaster. Stat92E-IR*	VDRC	VDRC_43867

Oligonucleotides		

Actin F: CACACCAAATCTTACAAAATGTGT	Eurofins Scientific	N/A
Actin R: AATCCGGCCTTGCACATG	Eurofins Scientific	N/A
upd2 F:	Eurofins Scientific	N/A
upd2 R:	Eurofins Scientific	N/A

Software and Algorithms		

FIJI (ImageJ)	https://ImageJ.net/Fiji/Downloads	Fiji-macOS
Adobe Illustrator	https://www.adobe.com/	Adobe Illustrator 2022
GraphPad Prism	https://www.graphpad.com/scientific-software/prism/	GraphPad Prism 9

## Highlights

- Fat and sugar regulate taste perception to avoid malnutrition, not caloric intake
- Sugar induces endocrine Hedgehog, which enhances fatty acid taste and suppresses sweet taste
- Fat induces Upd2 signaling, which suppresses fatty acid taste and increases sweet taste
- Fatty acid perception level is set by socs36E negative feedback in the taste neurons

## INCLUSION AND DIVERSITY

We support inclusive, diverse, and equitable conduct of research.
