# Supplementary material for: Fat- and sugar-induced signals regulate sweet and fat taste perception in Drosophila
Source: Cell Rep. Author manuscript; Available in PMC 2024 Jun 28. (PMC11212107; doi:10.1016/j.celrep.2023.113387)
Supplement: Supplemental [file NIHMS1967554-supplement-Supplemental.pdf]

Cell Reports, Volume *42*

Supplemental information

Fat- and sugar-induced signals regulate sweet and fat taste perception in *Drosophila*

Yunpo Zhao, Emilia Johansson, Jianli Duan, Zhe Han, and Mattias Alenius

Supplementary Figures

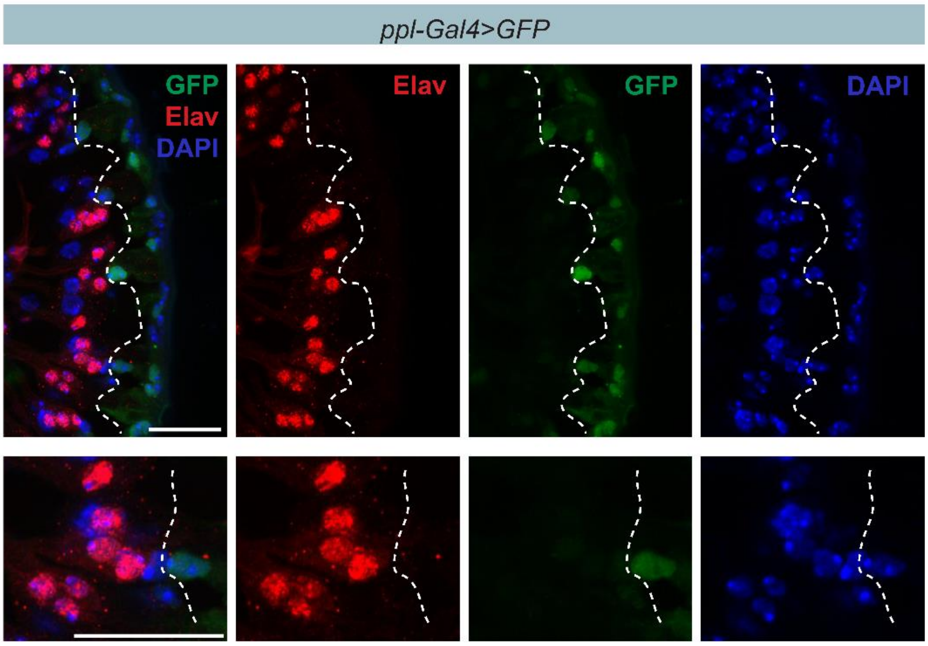

Supplementary Fig S1. *ppl-Gal4* is not expressed in the taste sensory neurons.

A representative confocal image of *ppl-Gal4>UAS-GFP* labellum. GFP is shown in green. Anti-Elav stains sensory neurons in red. DAPI stains DNA in blue. Scale bars represent 20 µm.

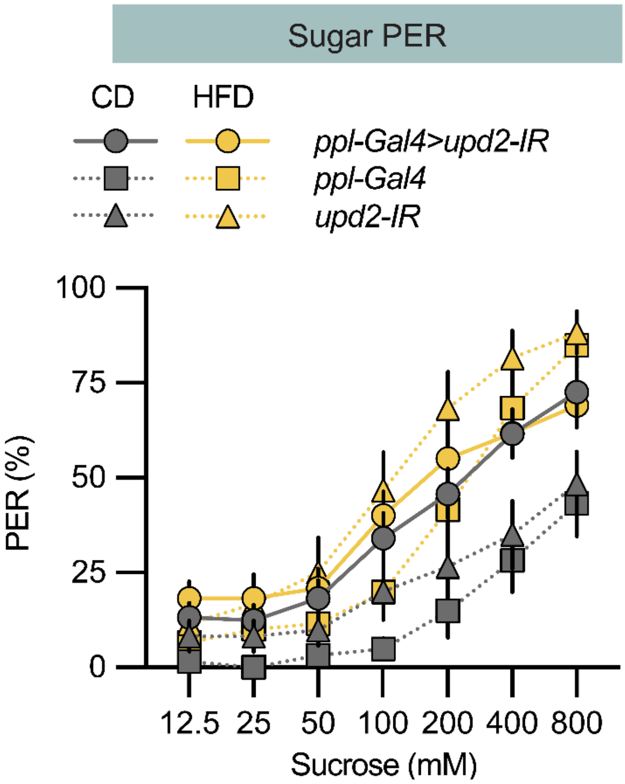

Supplementary Fig S2. Fat body derived Upd2 determines sugar sensation.

ppl-Gal4>upd2-IR (filleds) and control (*ppl-Gal4/+ and upd2-IR/+*, dotted lines) flies on control diet (CD, Grey) and high fat diet (HFD, yellow).

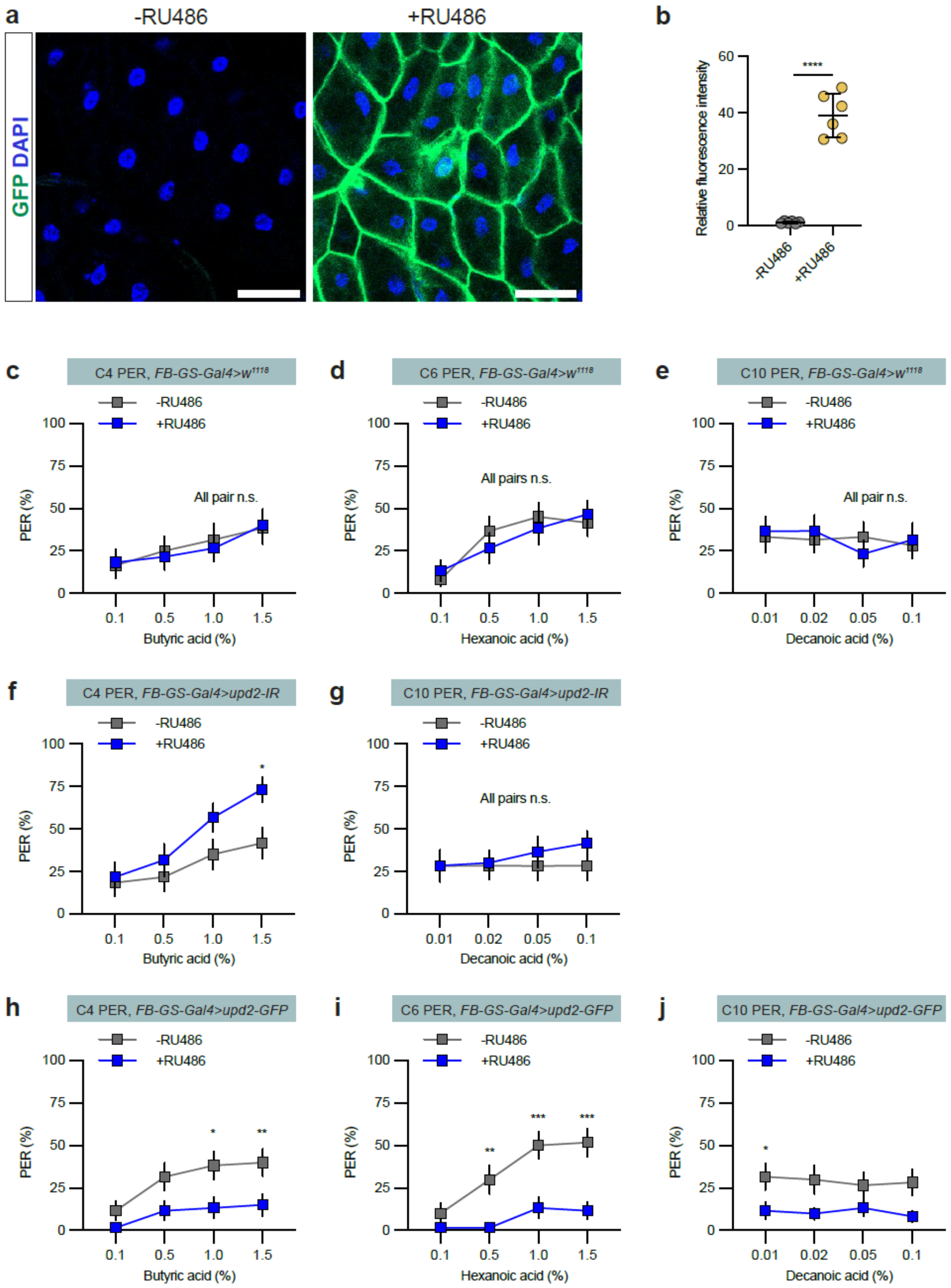

Supplementary Fig S3. Adipokine Upd2 regulates fatty acid sensation. (**a**) Representative confocal images of *FB-GS-Gal4>10xUAS-mCD8:GFP* without (left) or with RU486 4 days induction (right). GFP is shown in green. DAPI stains DNA in blue. Scale bars represent 20 µm. (**b**) Quantification of the relative fluorescence intensity of GFP in (**a**). *n* = 6 for each group. (**c-e**) RU486 does not change the PER of *FB-GS-Gal4>w^{1118}* flies in response to butyric acid (**c**), hexanoic acid (**d**), or decanoic acid (**e**) stimulations. *n* = 20 for each group. (**f**) Drug induced *FB-GS-Gal4>upd-IR* increases butyric acid PER. *n* = 20 for each group.(**g**) Drug induced *FB-GS-Gal4>upd-IR* does not have an effect on decanoic acid PER. *n* = 20 for each group. (**h-j**) RU486 induced *FB-GS-Gal>UAS-upd2-GFP* expression suppress PER to (**h**) butyric acid, (**i**) hexanoic acid, and (**j**) decanoic acid. *n* = 20 for each group. Data are presented as means ± SD (**b**) or SEM (**c-j**). Statistical analysis was performed via a two-tailed *t*-test (b) or Mann-Whitney tests (**c-j**). *, p<0.05; **, p<0.01; ***, p<0.001; ****, p<0.0001.

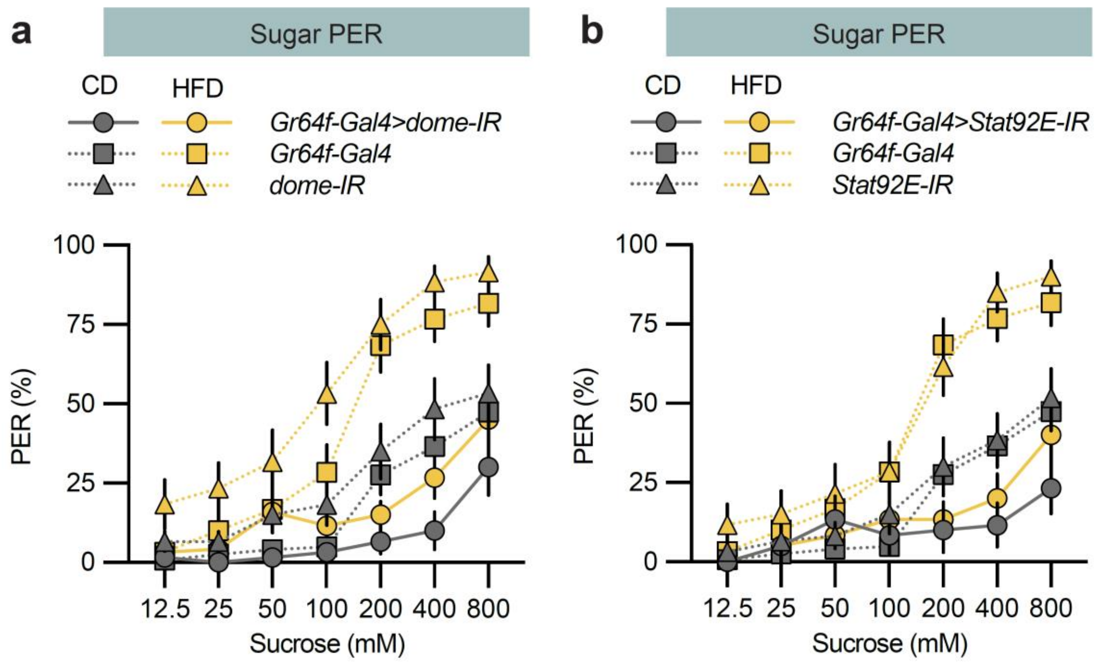

Supplementary Fig S4. Jak/STAT pathway in the sweet taste sensory neuron determines sugar sensation.

(**a**) *Gr64f-Gal4>dome-IR* (filled lines) and control (*Gr64f-Gal4/+* and *dome-IR*/+ dotted lines) flies on control diet (CD, Grey) and high fat diet (HFD, yellow). *n* = 20-40 flies for each group. (**b**) *Gr64f-Gal4>Stat92E-IR* (filled lines) and control (*Gr64f-Gal4/+* and *Stat92E-IR* dotted lines) flies on control diet (CD, Grey) and high fat diet (HFD, yellow). *n* = 20-40 flies for each group.

1L Food recipes	
Agar	10 g
Brewer's yeast	80 g
Yeast extract	20 g
Peptone	20 g
Sucrose	51 g Control and HFD, 342 g, 34% HSD
Propionic acid	6 mL
Nipagin	11 mL
Crisco (fat)	0 g Control and HSD, 141 g HFD

Supplementary Table 1 Fly food recipe
The basic fly food formula and a table with the amount added sugar and fat for each diet. HFD, high fat diet; HSD, high sugar diet
